# A Rare Case of a Pancreatic Intraductal Oncocytic Papillary Neoplasm Associated With Invasive Adenocarcinoma Presenting as a Gastric Mass

**DOI:** 10.7759/cureus.47886

**Published:** 2023-10-28

**Authors:** Ahmed I Younes, Xiaobang Hu, Lan Peng, Zhikai Chi

**Affiliations:** 1 Pathology, University of Texas Southwestern Medical Center, Dallas, USA

**Keywords:** intraductal oncocytic papillary neoplasm, adenocarcinoma, pancreas, pancreatic adenocarcinoma, pancreatic intraductal oncocytic papillary neoplasm

## Abstract

The World Health Organization recently recognized intraductal oncocytic papillary neoplasms of the pancreas (IOPNs) as distinct, pre-malignant pancreatic neoplasms. Due to their unique macroscopic and microscopic features, IOPNs are typically easy to diagnose and yield an indolent prognostic outcome. The diagnosis may be more complicated, and the prognosis may differ if an associated invasive carcinoma is present. Owing to the rarity of this entity, the available data is severely limited. Herein, we report a diagnostically challenging case of an IOPN associated with invasive carcinoma, initially presenting as a gastric mass with distinctive radiological and histopathological features.

## Introduction

From 1996 to 2019, intraductal oncocytic papillary neoplasms of the pancreas (IOPNs) were recognized by the World Health Organization as a subtype of intraductal papillary mucinous neoplasms (IPMNs) despite being previously reported as a distinct entity [1]. Thereafter, advances in molecular studies combined with its histopathological characteristics have led to the reidentification of IOPNs as a separate entity [2]. Macroscopically, IOPNs appear at least partially cystic and often multilocular. The papillary nodules are often not visible on gross examination despite being typically profuse under a microscope [3]. Histologically, IOPNs are characterized by arborizing papillae composed of mitochondrion-rich oncocytic cells. The cells that line these papillae are typically large, with well-defined borders, dense granular cytoplasm, and rounded nuclear borders. Their chromatin is homogeneous but relatively small, with prominent and slightly eccentric nucleoli [4]. IOPNs contain less intracytoplasmic mucin than IPMNs, complicating its differential diagnosis from solid adenocarcinomas upon radiological investigation. On cytological smears, the tumor cells exhibit a degree of uniformity with the formation of magenta-colored, thick, flat sheets. Blatant cellularity and striking architectural complexity are also commonly observed. The largest cohort reported to date included 24 patients with IOPNs; they had a slight female predominance and were more likely to develop the neoplasms in the pancreatic head or uncinate process [5].

Due to their unique macroscopic and microscopic features, IOPNs are typically easy to diagnose and yield an indolent prognostic outcome. The diagnosis may be more complicated, and the prognosis may differ if an associated invasive carcinoma is present. Owing to the rarity of this entity, the available data is severely limited. Herein, we report a diagnostically challenging case of an IOPN associated with invasive carcinoma, initially presenting as a gastric mass with distinctive radiological and histopathological features.

## Case presentation

**Figure 1:**
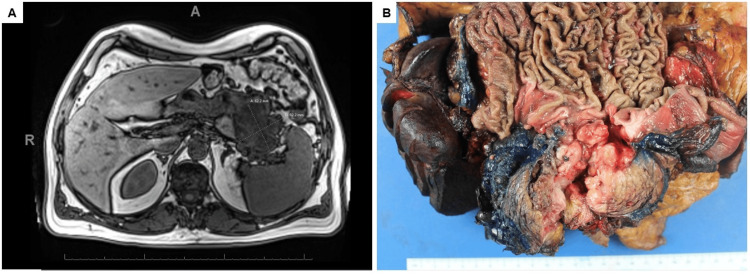
Imaging and gross examination of the mass. (A) Magnetic resonance imaging demonstrated an 8.0 cm mass that involved the pancreatic tail, gastric body, proximal jejunum, and splenic hilum. (B) Gross examination of the resected specimen revealed a soft, tan, homogeneous mass originating from the pancreas and involving the colon, stomach, small bowel, and spleen. A, anterior; R, right.

A 67-year-old man with a long smoking history underwent screening via chest computed tomography (CT), which revealed a large abdominal mass abutting the stomach and pancreas. CT of the abdomen revealed an 8 cm mass that was inseparable from the greater curvature of the stomach, tail of the pancreas, and splenic flexure of the colon. Magnetic resonance imaging (MRI) of the abdomen confirmed the presence of a mucinous mass involving the pancreatic tail, gastric body, proximal jejunum, and splenic hilum (Figure 1). Esophagogastroduodenoscopy (EGD) revealed abundant mucin covering a large gastric mass, resembling a waterfall. The patient had a past history of post-cholecystectomy pancreatitis with pseudo-cyst formation, which necessitated a pancreatic cystogastrostomy for drainage. Therefore, the mass was considered to represent a primary gastric tumor extending into the pre-existing cystogastrostomy drain/diverticulum.

**Figure 2:**
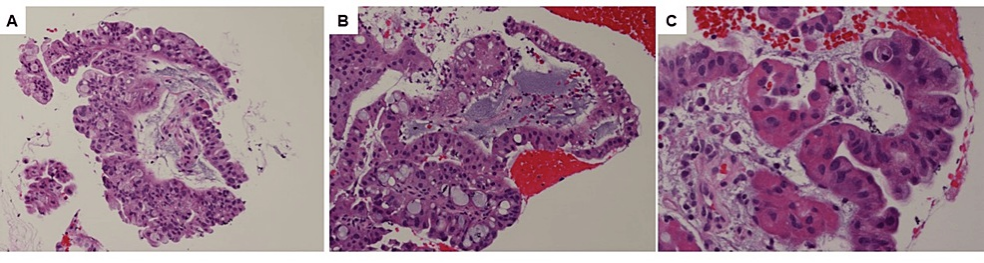
Histology for the gastric mass from fine-needle aspiration cell block before neoadjuvant chemotherapy. (A) Adenocarcinoma with papillary architecture (hematoxylin and eosin [H&E], 200×). (B) Adenocarcinoma with mucinous features (H&E, 200×). (C) Adenocarcinoma with oncocytic features (H&E, 400×).

Fine-needle aspiration biopsy was performed on the gastric mass by sampling through the gastric wall. The cell block indicated an adenocarcinoma with papillary architecture, mucinous, and oncocytic features (Figure 2A, 2B). The carcinoma cells were cuboidal or columnar with eosinophilic granular cytoplasm and large, round nuclei, some with prominent nucleoli, and forming arborizing papillae with delicate fibrovascular cores. Dispersed goblet tumor cells can be seen (Figure 2C). Due to limited specimen in the cell block, no definitive immunohistochemical workup was conducted. The patient subsequently received eight cycles of neoadjuvant chemotherapy (FOLFIRINOX - leucovorin calcium, fluorouracil, irinotecan hydrochloride, and oxaliplatin) before undergoing en bloc resection of the mass with partial stomach, distal pancreas, spleen, partial small bowel, and partial colon. Gross examination of the resected specimen revealed that the distal pancreatic parenchyma was replaced with a friable papillary mass (8.3 × 6.3 × 4.5 cm) that appeared to originate from the main pancreatic duct and involved the colon, stomach, small bowel, and spleen. The mass communicated with the colon and stomach through a fistula-tract-like formation, with a transmural perforation of the stomach (Figure 1). The tumor extended to the superior pole of the spleen. Sectioning revealed a soft, tan, homogeneous cut surface with a necrotic, hemorrhagic central cavity.

**Figure 3:**
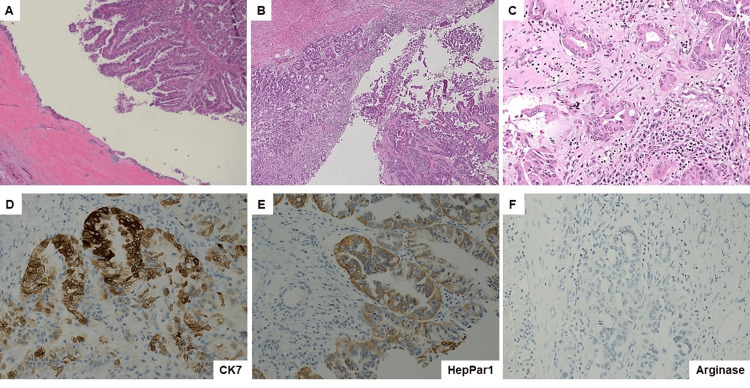
Histology and immunostaining for the tumor involving stomach from resection specimen (A) Low-power view of invasive carcinoma extending through the prior pancreatic cystogastrostomy/fistula-tract-like formation (hematoxylin and eosin [H&E], 40×). (B) Low-power view of invasive carcinoma in relationship with unremarkable gastric mucosa (H&E, 40×). (C) High-power view of carcinoma cells invading gastric wall with a severe desmoplastic stromal response (H&E, 200×). (D-F) The invasive carcinoma cells stained diffusely positive for (D) cytokeratin 7 (CK7, 200×) and (E) HepPar1 (200×) but negative for (F) arginase (200×).

**Figure 4:**
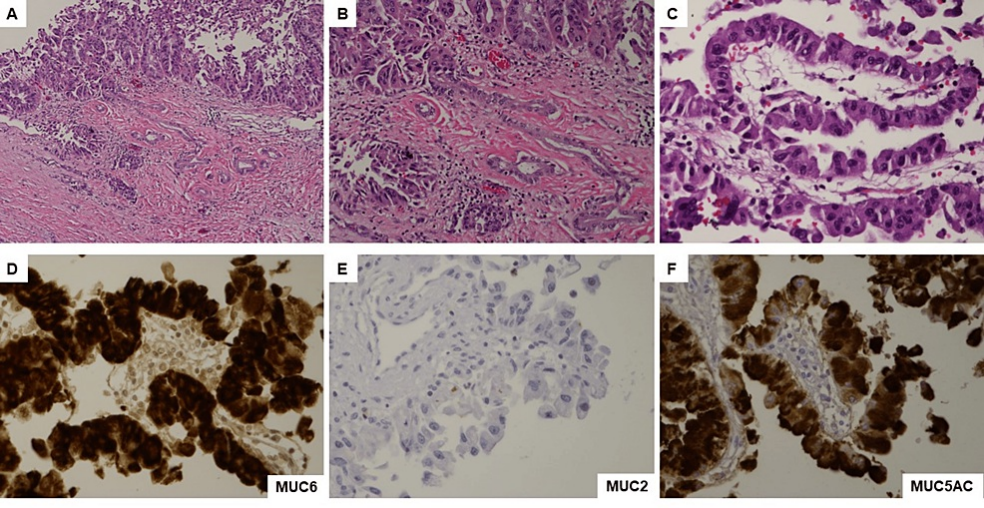
Histology and immunostaining for the tumor involving pancreas from resection specimen. (A) Invasive carcinoma arose from an intraductal oncocytic papillary neoplasm (IOPN) of the pancreatic tail with characteristic papillary configuration and oncocytic cells (hematoxylin and eosin [H&E], 100×) and (B) high-power view (H&E, 200×). (C) High-power view of IOPN cells with dense granular cytoplasm, papillary architecture, and high-grade dysplasia including prominent nucleoli, nuclear enlargement, and loss of polarity (H&E 400×). The IOPN cells are positive for (D) MUC6 (400×), negative for (E) MUC2 (400×), and positive for (F) MUC5AC (400×).

Microscopic examination revealed that the tumor cells had invaded the gastric mucosa with no gastric precursor lesions identified. Similar to the cytology findings, the carcinoma cells were cuboidal or columnar with eosinophilic granular cytoplasm and large, round nuclei, some with prominent nucleoli forming papillary architectures (Figure 3). Many interspersed goblet cells were observed. A severe desmoplastic stromal response was also observed. The carcinoma cells were diffusely positive for cytokeratin 7 (CK7), epithelial membrane antigen (EMA), hepatocyte paraffin 1 monoclonal antibody (HepPar1), MUC5AC, and MUC6, sparsely positive for synaptophysin, and negative for arginase, chromogranin, cytokeratin 20, CDX2, Pax8, trypsin, chymotrypsin, and MUC2. Interestingly, sections of the pancreatic portion of the specimen confirmed that the invasive carcinoma arose from a pancreatic IOPN and extended directly through the prior pancreatic cystogastrostomy/fistula-tract-like formation into the stomach, as well as directly invading the small bowel and colon (Figure 4). The IOPN cells were also positive for EMA, HepPar1, MUC6, and MUC5AC, but negative for arginase or MUC2. Beta-catenin exhibited membranous staining, whereas mucicarmine revealed intracellular mucin vacuoles. Twenty-five regional lymph nodes were identified, all negative for metastatic carcinoma cells. All surgical resection margins were negative for IOPN and carcinoma. Neoadjuvant chemotherapy resulted in residual cancer with evident tumor regression (a partial response [score = 2]). No lymphovascular or perineural invasions were observed. The final pathological stage was ypT3N0. The patient was clinically stable seven months after surgery and exhibited no disease recurrence without receiving adjuvant therapy.

## Discussion

Pancreatic cancer is the fourth leading cause of cancer-related mortality in the United States. The advanced disease state of patients at the time of diagnosis remains a formidable barrier to treatment [6]. Although the spread of pancreatic cancer to the stomach typically occurs in the context of an extensive occult malignancy, gastric involvement can occur via a variety of other mechanisms, including hematogenous dissemination, lymphatic metastasis, intramural or intraluminal dissemination, and intraoperative seeding [7]. Therefore, the origin of the primary pancreatic carcinoma has to be determined in relation to the stomach via imaging studies and endoscopic ultrasound. The oncologist also has to rule out the possibility of direct tumor infiltration or the presence of multiple primary malignancies. Furthermore, in a small number of cases, primary pancreatic cancer originates from heterotopic pancreatic tissue in the stomach [8].

In contrast, in our case, the tumor originated from an IOPN. IPMNs and IOPNs are characterized by papillary proliferation of epithelial cells, cystic dilation, and mucin secretion. They range from adenoma-like masses to invasive cancers with varying degrees of aggression [9]. The pancreatic parenchyma and regional lymph nodes can be infiltrated by the IPMN/IOPN without the presence of invasive adenocarcinoma as both benign and malignant forms can penetrate neighboring organs [10]. Spontaneous perforation of the tumor due to increased intraductal pressure resulting from mucin secretion has been proposed as a potential mechanism for the penetration of IPMNs/IOPNs into adjacent organs. Patients with an IPMN/IOPN that has invaded other organs have a five-year survival rate of only 28%, which is worse than that in patients without local invasion [11]. Invasive pancreatic cancers arising from IPMNs/IOPNs may respond to adjuvant therapy; however, current data suggest that it should be selectively administered based on individual tumor characteristics [12].

In our case, CT and MRI revealed an enormous solid and cystic mass that had directly invaded the stomach. EGD revealed the unusual presence of abundant mucin covering an exceptionally large mass, resembling a waterfall. The final diagnosis was validated via pathological analysis of the resected specimen. Despite the higher-than-usual mucin production, invasive carcinoma cells retained the morphological features of IOPN cells. The IOPN cells in our case are positive for MUC6 and HepPar1 immunostains, but negative for MUC2 and arginase immunostains. The main differential diagnosis for our case is the intestinal subtype of IPMN with associated carcinoma. However, the intestinal subtype of IPMN cells should be negative for MUC6, but positive for MUC2 immunostains, while both IOPN and IMPN cells are positive MUC5AC immunostains [2]. In addition, the tumor cells from fine-needle aspiration cell block, which are before neoadjuvant chemotherapy, show similar morphological features to those from resection specimens. Taken together, the overall morphological and immunohistochemical features in our case would be consistent with IOPN-associated invasive adenocarcinoma. A previously reported case demonstrated that IOPN may be a source of invasive colloid carcinoma [4]. By contrast, our case demonstrated the presence of a solid mucinous tumor other than colloid carcinoma, before and after neoadjuvant chemotherapy. When EGD reveals mucin cascades in the gastrointestinal tract, the oncologist has to consider the possibility of pancreatic cancer infiltration into the adjacent organs, and additional imaging modalities are required to confirm the diagnosis. As the tumor epicenter was located in the pancreas and given its unique morphological features, distinct immunohistochemical profiles, and the presence of precursor lesion (IOPN), we concluded that this was a primary pancreatic tumor with direct invasion of surrounding organs, probably through the pre-existent pancreatic cystogastrostomy drainage site.

In our case, the differential diagnoses also included primary gastric adenocarcinoma and a pancreatic neuroendocrine tumor (PanNET). Certain variants of gastric adenocarcinoma exhibit oncocytic differentiation, similar to pancreatic adenocarcinoma, but they can be distinguished by the presence of dysplastic gastric mucosa as a precursor lesion [13]. In addition, pancreatic adenocarcinomas (especially those exhibiting oncocytic features) can resemble PanNETs in some instances, but they are usually negative for neuroendocrine markers, such as synaptophysin and chromogranin, as in this case [14]. We also considered the possibility of a solid pseudopapillary neoplasm of the pancreas; however, nuclear staining for beta-catenin was negative [15].

Recent advances in our understanding of molecular pathogenesis have led to the development of immunoprofiles that distinguish IOPNs from ductal adenocarcinoma and IPMNs. Previously, immunoprofiles were determined for 24 IOPNs and 22 IPMNs, revealing that MUC2 and MUC6 expression differed between them [2]. In addition, the genomic landscapes of IPMNs and IOPNs differ, with IOPNs lacking KRAS and GNAS mutations, exhibiting substantially higher APC mutation rates, and displaying the presence of fusion genes involving the PRKACA and PRKACB genes [4,16]. Sequencing of the IOPN transcriptome revealed increased transcriptional activity of the DNAJB1 and ATP1B1 promoters, which induced overexpression of the PRKACA and PRKACB genes, leading to increased protein kinase A (PKA) activity and subsequent mitochondrial hyperplasia [17]. This may be the biochemical basis for the cytological and histological similarities between fibrolamellar hepatocellular carcinomas and IOPNs, as PKA is active in both tumor types.

## Conclusions

In summary, we reported a rare case of an IOPN resulting in invasive pancreatic cancer, initially presenting as a large gastric mass. There is limited literature on IOPN cases with invasive adenocarcinoma, especially those with extensive invasive components. To the best of our knowledge, there have been no reported cases of IOPN presenting as a gastric mass. In addition, our case contributes to the literature on IOPNs associated with invasive adenocarcinoma, exhibiting marked mucinous characteristics. Primary gastric adenocarcinoma is a major differential diagnosis that must be excluded. Therefore, a definitive diagnosis requires a comprehensive evaluation of clinical history as well as radiological, endoscopic, histopathological, and immunohistochemical examinations.
